# Increased gene expression of CCR6 and RORγt in peripheral blood cells of rheumatoid arthritis patients and their correlation with anti‐cyclic citrullinated peptide and disease activity

**DOI:** 10.1002/iid3.1112

**Published:** 2023-12-06

**Authors:** Seyed Askar Roghani, Ramin Lotfi, Masood Ghasemzade Soroush, Ali Khorasanizadeh, Parisa Feizollahi, Shirin Assar, Parviz Soufivand, Mehran Pournazari, Zahra Mohammadi Kish, Mahdi Taghadosi

**Affiliations:** ^1^ Student Research Committee, Medical School Kermanshah University of Medical Sciences Kermanshah Iran; ^2^ Immunology Department, Faculty of Medicine Kermanshah University of Medical Sciences Kermanshah Iran; ^3^ Medical Biology Research Center, Health Technology Institute Kermanshah University of Medical Sciences Kermanshah Iran; ^4^ Clinical Research Development Center, Tohid Hospital Kurdistan University of Medical Sciences Sanandaj Iran; ^5^ Lung Diseases and Allergy Research Center, Research Institute for Health Development Kurdistan University of Medical Sciences Sanandaj Iran; ^6^ Student Research Committee, School of Medicine Ahvaz Jundishapur University of Medical Sciences Ahvaz Iran; ^7^ Clinical Research Development Center, Imam Reza Hospital Kermanshah University of Medical Sciences Kermanshah Iran

**Keywords:** anti‐CCP, CCR6, DAS‐28, rheumatoid arthritis, RORγt

## Abstract

**Objectives:**

The significance of T helper 17 (Th17) cells in the pathogenesis of rheumatoid arthritis (RA) has recently been demonstrated in many studies. Retinoic acid receptor‐related orphan receptor γt (RORγt) is a transcription factor that is specifically involved in the generation of Th17 cells. Besides, the chemokine receptor CCR6, the receptor for CCL20, is characteristically expressed by these cells. Considering the pivotal roles of Th17 cells in RA pathogenesis, in this study, we assessed the gene expression of CCR6 and RORγt in the peripheral blood leukocytes of new case RA patients. Also, we evaluated their association with anticyclic citrullinated peptide (anti‐CCP) antibodies and disease activity.

**Methods:**

Forty‐five new case RA patients and 45 healthy persons have been recruited in this investigation. The gene expression of CCR6 and RORγt was evaluated by quantitative real‐time PCR (qRT‐PCR), and anti‐CCP antibodies plasma levels were measured using the enzyme‐linked immunosorbent assay (ELISA) technique. Disease activity was measured according to the disease activity score‐28 (DAS‐28) formula.

**Results:**

The gene expression of CCR6 and RORγt increased remarkably in new case RA patients compared to healthy controls (*p* < .05 and *p* < .01, respectively). Moreover, there was a positive correlation between RORγt gene expression and parameters, including gene expression of CCR6 (*p* = .001, *r* = .461), plasma levels of CCL20 (*p* = .0009, *r* = .477), ESR (*p* = .004, *r* = .419), DAS‐28 (*p* = .006, *r* = .402), anti‐CCP (*p* = .019, *r* = .346), and RF (*p* = .001, *r* = .451). Also, CCR6 gene expression was positively associated with the DAS‐28 (*p* = .037, *r* = .310), plasma levels of anti‐CCP (*p* = .037, *r* = .312), and ESR (*p* = .029, *r* = .327).

**Conclusion:**

Increased gene expression of CCR6 and RORγt in peripheral blood leukocytes of new case RA patients may contribute to the exacerbation and pathogenesis of RA.

## Graphical abstract

The gene expression of CCR6 and RORγt increased remarkably in new case RA patients compared to healthy controls. Moreover, there was a positive correlation between RORγt gene expression and parameters, including gene expression of CCR6, plasma levels of CCL20, ESR, DAS‐28, anti‐CCP, and RF. Also, CCR6 gene expression was positively associated with the DAS‐28, plasma levels of anti‐CCP, and ESR. Increased gene expression of CCR6 and RORγt in peripheral blood leukocytes of new case RA patients may contribute to the exacerbation and pathogenesis of RA

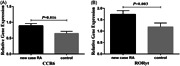

## 1 INTRODUCTION

Rheumatoid arthritis (RA) is a complex chronic immune‐mediated inflammatory disease (IMID) that mainly affects the small joints.^1^ RA, as a genetically, pathologically, and clinically heterogeneous disorder with different subtypes, affects almost 1% of the global people.^2^ Various molecules, including autoantibodies, major histocompatibility complex (MHC), pro‐inflammatory cytokines, and chemokines, play a crucial role in the pathogenesis of RA.^3^, ^4^, ^5^, ^6^ Chemokines and chemokine receptors have an indispensable role in leukocyte migration and extravasation in RA.^7^ Chemokine receptors have been classified into two large groups, including CCR and CXCR groups, according to their amino acid cysteine.^8^ Multiple chemokines and their receptors, including cysteine–cysteine motif chemokine ligand 2 (CCL2), CCL3, CCL4, CXCL10, CCR1, CCR2, and CCR5, have been implicated in the pathogenesis of RA.^7^, ^9^ More recently, we have reported a highly significant elevation in the plasma levels of CCL20 in newly diagnosed RA patients, which was in association with laboratory and clinical parameters of RA.^10^ CCL20 mediates its effect by binding to the CCR6, which is mainly expressed by T helper 17 (Th17) cells; cells that not only fulfill a crucial role in protecting the host against fungal and bacterial infections but also in multiple immune‐mediated inflammatory disorders, comprising RA, inflammatory bowel disease, uveitis, multiple sclerosis, asthma, and psoriasis.^11^, ^12^, ^13^ A previous study showed that the CCR6–CCL20 axis has important effects on RA pathogenesis.^14^ CCR6 mediates the ingress of Th17 cells into inflamed synovial tissues in RA.^7^ Furthermore, using a genome‐wide association study (GWAS) of RA, it was revealed that the polymorphism of the CCR6 gene is linked with susceptibility to RA.^15^ It is worth considering that the development, differentiation, and function of Th17 cells depend on the retinoic acid receptor‐related orphan receptor γt (RORγt), which is the Th17 lineage‐specific transcription factor. Also, RORγt, which is encoded by the *RORC* gene, enhances the interleukin‐17 (IL‐17) generation, and as a result, enhances the immune cells' attraction and migration to the sites of inflammation.^16^, ^17^ Given the existing data about the importance of CCR6 and RORγt in the pathogenesis of RA, in the present investigation, we assessed the gene expression of CCR6 and RORγt in peripheral blood leukocytes of new case RA patients compared to healthy individuals. To survey the impact of CCR6 and RORγt gene expression on RA severity, we analyzed their association with Disease Activity Score‐28 (DAS‐28), the well‐established criteria of RA disease activity, and anti‐cyclic citrullinated peptide (anti‐CCP), a strong serologic predictor of RA severity.^18^

## 2 MATERIALS AND METHODS

### 2.1 Study population

The current cross‐sectional investigation has been performed on 45 new case RA patients who were referred to Imam Reza Hospital, Kermanshah University of Medical Sciences (KUMS), Kermanshah, Iran. Besides, 45 age‐ and gender‐matched healthy subjects lacking any background of autoimmune diseases were chosen as the control group. The study was per the Declaration of Helsinki and was performed with approval from the Ethics Committee of the KUMS (Ethical code: IR.KUMS.REC.1400.175). All the patients and control population signed informed consent. Meanwhile, all the patients were diagnosed according to the American College of Rheumatology/European League Against Rheumatism (ACR/EULAR) 2010 classification criteria for RA^19^ by an expert rheumatologist.

### 2.2 RNA isolation and cDNA synthesis

Total RNA was extracted from whole blood specimens according to the manufacturer's instructions (RNX plus, SinaClon). The concentration and purity of the extracted RNA were measured using a NanoDrop 2000 UV–Vis Spectrophotometer (Thermo Scientific), and further, its purity was evaluated by 1% agarose gel electrophoresis. The extracted RNA was aliquoted and stored at −70°C until use. Complementary DNA (cDNA) synthesis was conducted using the Easy cDNA Synthesis Kit (Parstous Biotechnology) according to the manufacturer's guidelines.

### 2.3 Real‐time PCR for CCR6 and RORγt genes

Primer designing for genes of CCR6, RORγt, and GAPDH, as an internal control for normalization, was accomplished using online software, including Oligocalc and Oligoanalyzer. The oligonucleotide sequences of the designed primers and their PCR product length are represented in Table 1. The accuracy and specificity of the designed primers were confirmed using the Basic Local Alignment Search Tool on the US National Center for Biotechnology Information (NCBI) website (http://www.ncbi.nlm.nih.gov/tools/primer-blast/). Real‐time PCR analysis was accomplished in a final volume of 15 µL containing 1 μL of cDNA, 0.5 mM of each forward and reverse primer, 7.5 μL of PCR Master Mix (Parstous Biotechnology), and 5.5 μL of ddH_2_O. The PCR reactions were carried out on the Light cycler 96 (Roche) using the thermal cycling parameters (95°C 30 s, 40 cycles of 5 s at 95°C, 30 s at 60°C, 62°C, and 65°C for GAPDH, CCR6, and RORγt, respectively; melting curve: 5 s at 95°C, 15 s at 65°C, 5 s at 95°C, and continues melting). All samples were run in duplicates to diminish the risk of false‐positive or false‐negative results owing to technical errors. The relative quantity of target mRNA in samples was computed and normalized to the corresponding GAPDH mRNA transcript level as a housekeeping gene. The relative gene expression for each sample was determined using the following formula (ratio = (*E*_target_)^ΔCt target (control‐sample)^/(E_Ref_)^ΔCt Ref (control‐sample)^), as formerly described by Pfaffl.^20^

**Table 1:** Oligonucleotide sequence of the designed primers for qRT‐PCR amplification.

Gene name	Forward primer (5′–3′)	Reverse primer (5′–3′)	PCR product length (bp)
GAPDH	GAAACCTGCCAAGTATGATG	AGGAAATGAGCTTGACAAAG	188
CCR6	CCACAATGAGCGGGGAATCAATGAA	CAAATAGCCTGGAGAACTGCCTGAC	150
RORγt	CCTGCTGAGAAGGACAG	GATCCCAGACGACTTGTC	109

### 2.4 Measurement of anti‐CCP and CCL20 plasma levels

The plasma levels of anti‐CCP and CCL20 have been measured using the enzyme‐linked immunosorbent assay (Medizym and Eastbiopharm, respectively) based on the manufacturer's recommendations.

### 2.5 DAS‐28

The disease activity score was evaluated by an expert rheumatologist according to the formula DAS28 = 0.56 (TJ)^½^ + 0.28 (SJ)^½^ + 0.70 ln (ESR) + 0.014 GH (TJ: number of tender joints from 28 joints, SJ: number of swollen joints from 28 joints, GH: global health, ESR: erythrocyte sedimentation rate), as described previously by Inoue et al.^21^

### 2.6 Statistical analysis

Statistical analysis was performed with SPSS software (version 21.0, SSPS Inc.) and the software GraphPad Prisms® 6.0 (GraphPad Software). The 1‐sample Kolmogorov–Smirnov test (1‐sample K–S test) determined the distribution normality in the groups. The *p* value is based on the independent *t*‐test or Mann–Whitney nonparametric test for statistical comparisons between both RA and control groups. The correlation between the two variables was determined by the Spearman rank correlation analysis. The *p* value is considered statistically significant at the level of <.05.

## 3 RESULTS

### 3.1 Studied participants

The current study investigated 45 new case RA patients and 45 healthy persons. In terms of age and sex, there was no significant difference between new case RA patients and controls (*p* > .05). Moreover, the mean BMI was significantly greater in the new case RA patients when compared to the controls (*p* < .05). The demographic information and clinical properties of participants are shown in Table 2.

**Table 2:** The demographic and clinical information of the studied participants.

Variables	RA patients (*N* = 45)	Healthy controls (*N* = 45)	*p* Value
Age (years)	48.84 ± 1.68	48.98 ± 1.82	.957
Sex (F/M)	32/13	32/13	
BMI (kg/m^2^)^a^	26.83 ± 0.59	25.07 ± 0.57	.027
CCL20 plasma levels (ng/L)	305.94 ± 17.21	138.35 ± 6.31	<.001
AntiCCP‐IgG (U/mL)	619.84 ± 55.35	/	
RF (IU/mL)	115.73 ± 9.47	/	
ESR (mm/h)	26.91 ± 2.81	/	
Swollen joint	2.11 ± 0.22	/	
Tender joint	3.82 ± 0.40	/	

*Note*: All data are expressed as the mean value ± SEM.

Abbreviations: BMI, body mass index; ESR, erythrocyte sedimentation rate; N, number; RF, rheumatoid factor.

^a^ BMI was calculated for each individual as weight in kilograms divided by the squared height in meters (kg/m^2^).

### 3.2 The gene expression levels of CCR6 and RORγt in peripheral blood cells of study groups

**Figure 1:**
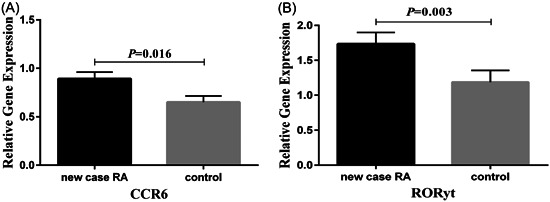
Gene expressions analysis. (A) The expression levels of CCR6 significantly increased in the newly diagnosed rheumatoid arthritis patients compared to the control group. (B) RORγt gene expression in the new case group was significantly higher than that of controls.

The gene expression of CCR6 and RORγt increased significantly in peripheral blood leukocytes of the new case RA patients compared to healthy subjects (*p* = .016 and *p* = .003, respectively) (Figures 1A,B, in order).

### 3.3 The plasma concentrations of CCL20 in patients and controls

**Figure 2:**
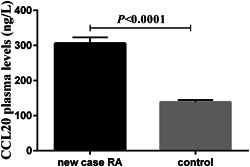
Comparing the plasma concentrations of cysteine–cysteine motif chemokine ligand 20 (CCL20) in the new case rheumatoid arthritis (RA) patients and healthy controls. The plasma concentrations of CCL20 in the new case RA patients were significantly higher than in controls.

The mean plasma concentrations of CCL20 were significantly elevated in the new case RA patients, in comparison with the healthy controls (*p* < .0001) (Figure 2).

### 3.4 Correlation of the RORγt gene expression with the laboratory and clinical parameters

**Figure 3:**
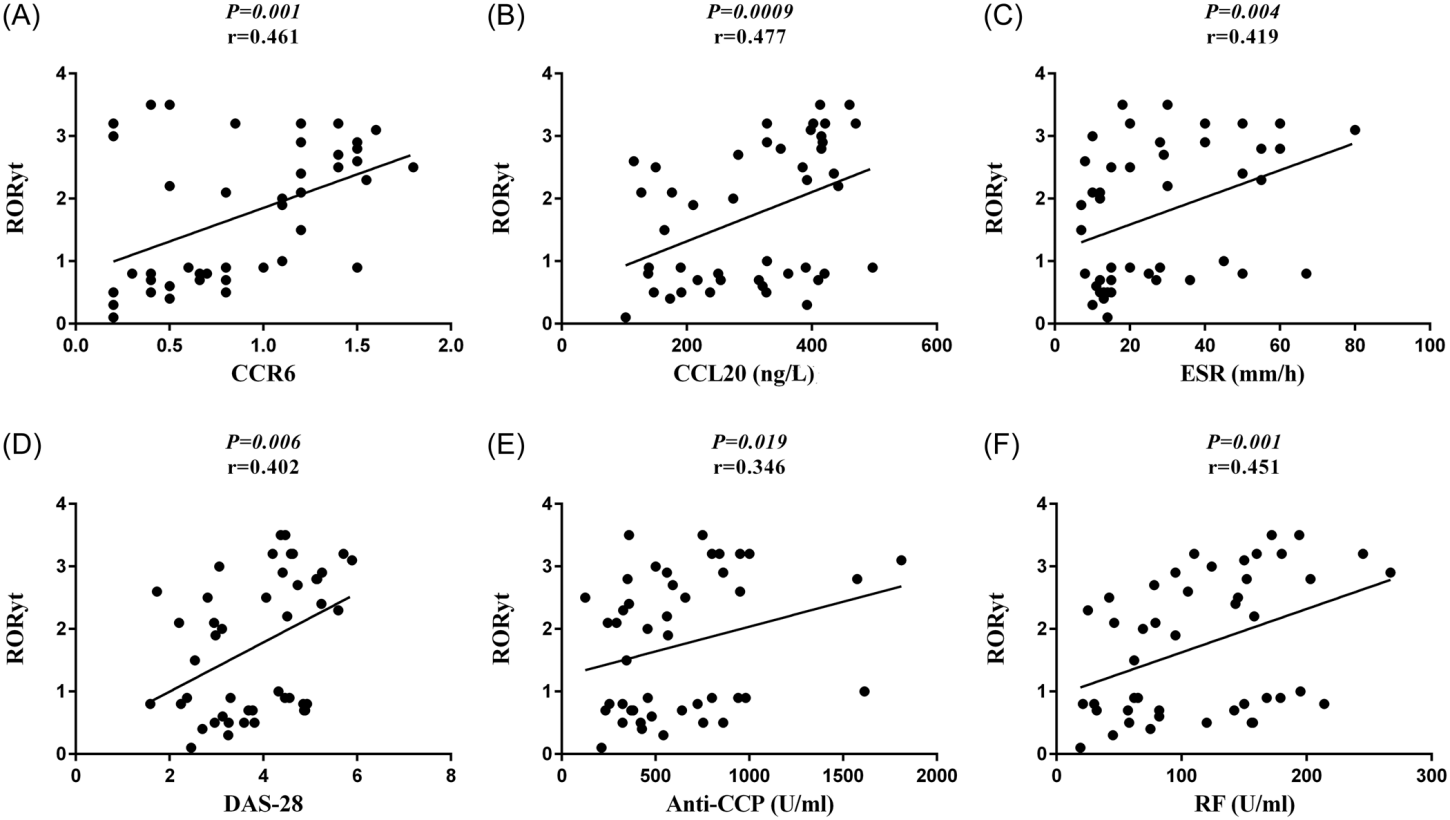
Correlation of RORγt with clinical and laboratory parameters in the new case RA patients. (A) RORγt gene expression was positively correlated with the CCR6 gene expression in the new case RA patients (*p* = .001, *r* = .461). (B) There was a positive association between cysteine–cysteine motif chemokine ligand 20 (CCL20) plasma level and RORγt gene expression in the new case RA patients (*p* = .0009, *r* = .477). (C) The gene expression of RORγt was positively associated with the erythrocyte sedimentation rate in the new case RA patients (*p* = .004, *r* = .419). (D) There was a positive relationship between RORγt gene expression and Disease Activity Score‐28 in the new case RA patients (*p* = .006, *r* = .402). (E) RORγt gene expression was positively correlated with the anti‐CCP plasma levels in the new case RA patients (*p* = .019, *r* = .346). (F) The shown graph depicts a positive correlation between the RORγt gene expression and rheumatoid factor in the new case RA patients (*p* = .001, *r* = .451).

There was a positive correlation between gene expression of RORγt and parameters, including CCR6 gene expression (*p* = .001, *r* = .461), CCL20 plasma levels (*p* = .0009, *r* = .477), ESR (*p* = .004, *r* = .419), DAS‐28 (*p* = .006, *r* = .402), anti‐CCP plasma levels (*p* = .019, *r* = .346), and rheumatoid factor (RF) (*p* = .001, *r* = .451), in the newly diagnosed RA patients (Figure 3–f, respectively).

### 3.5 Correlation of the CCR6 gene expression with the laboratory and clinical parameters

**Figure 4:**
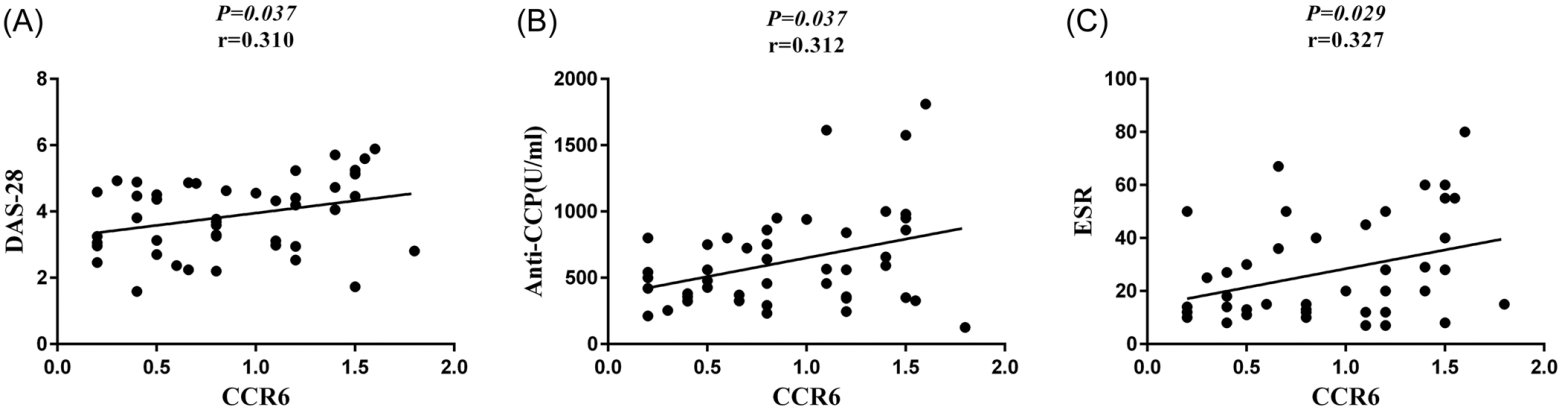
Correlation of the CCR6 gene expression with the Disease Activity Score‐28 (DAS‐28) and anti‐cyclic citrullinated peptide (anti‐CCP) in the new case RA patients. (A) The gene expression of CCR6 was positively correlated with the DAS‐28 in the new case RA patients (*p* = .037, *r* = .310). (B) There was a positive association between anti‐CCP plasma level and CCR6 gene expression in the new case RA patients (*p* = .037, *r* = .312). (C) CCR6 gene expression was positively associated with the ESR in the new case RA patients (*p* = .029, *r* = .327).

The gene expression of CCR6 was positively correlated with the DAS‐28 (*p* = .037, *r* = .310), plasma levels of anti‐CCP (*p* = .037, *r* = .312), and ESR (*p* = .029, *r* = .327) (Figure 4a–c, in order).

## 4 DISCUSSION

There are several studies in which the importance of Th17 cells has been indicated in RA pathogenesis.^22^, ^23^, ^24^, ^25^ In this study, we assessed the gene expression of CCR6, the chemokine receptor characteristically expressed by the Th17 cells, and RORγt, the master regulator transcription factor for these cells, in the peripheral blood cells of new case RA patients compared to the healthy controls, as well as their correlation with the anti‐CCP and disease activity.

The gene expression of CCR6 was significantly elevated in the newly diagnosed RA patients compared to the controls in our investigation. CCR6 is classified in the CC chemokine receptor group and is mainly expressed by memory T cells and dendritic cells.^26^ CCR6 plays a key role in health and disease and also has an indispensable role in the recruitment of Th17 cells, the herald cells in inflammatory reactions.^27^, ^28^ In line with our findings, the previous studies showed that a polymorphism in CCR6 increases the susceptibility to RA.^14^, ^29^ Also, CCR6 has been shown to have an important impact on the pathogenesis of psoriatic arthritis (PsA), and targeting CCR6 could have a promising effect in the treatment of PsA.^28^

CCL20, the exclusive ligand for CCR6, is produced by Th17 cells.^30^ More recently, we reported elevated levels of CCL20 in the plasma of newly diagnosed RA patients.^10^ Regarding these findings in the newly diagnosed RA patients, it can be concluded that the CCR6‐CCL20 interaction may underpin early pathological events in RA. The study conducted by Hirota et al. (2007) confirmed the importance of CCR6‐CCL20 in the pathogenesis of synovitis in animal models.^30^ In our study, there was a positive correlation between CCR6 gene expression in peripheral leukocytes and DAS‐28. This finding shows that CCR6 might be associated with RA disease severity.

In line with this finding, Razavy et al. study recently showed that the IL23R (GFP)^+^ CD4^+^ T cells that express CCR6 are present in the inflamed joints in early arthritis.^31^ In the following, we found a positive correlation between the CCR6 gene expression and the anti‐CCP plasma level. A previous study indicated that anti‐CCP is an indicator of RA disease activity.^32^ This finding highlights the possible effects of CCR6 gene expression on RA disease activity in the new case RA patients. Recently, Kaneko et al. (2018) showed that the CCR6 expression is considerably higher in Th17 cells from peripheral blood mononuclear cells (PBMCs) of RA patients, in comparison to osteoarthritis patients and healthy controls,^33^ which this study is in line with our findings. Altogether, targeting the CCR6‐CCL20 axis by using neutralizing monoclonal antibodies or small‐molecule antagonists might become a promising therapeutic approach to control and reduce the severity of autoimmune reactions in the coming years.^34^

RORγt, a ligand‐dependent transcription factor belonging to the superfamily of the nuclear hormone receptor (NHR), is the Th17 cell‐specific transcription factor regulating the transcription of IL‐17, an inflammatory cytokine.^16^, ^35^, ^36^ In the present study, the gene expression of RORγt was strikingly enhanced in peripheral blood cells of new case RA patients when compared to healthy controls. Consistent with our findings, another study indicated that the expression of RORγt is significantly increased in Th17 cells from PBMCs of RA patients relative to osteoarthritis patients and healthy controls.^33^ Furthermore, overexpression of RORγt in T cells has been found to protect against begetting collagen‐induced arthritis, the animal model of autoimmune arthritis.^37^ Noteworthy, the study of Hirota et al. (2007) showed that RORγt regulates the expression of CCR6.^30^ These findings raise the possibility that increased gene expression of RORγt induced the enhancement of CCR6 gene expression, leading to the migration and then agglomeration of Th17 cells in the inflamed joints of patients with RA. Additionally, in our study, the gene expression of RORγt was positively associated with the parameters, including CCR6 gene expression, CCL20 plasma levels, ESR, DAS‐28, anti‐CCP plasma levels, and RF, in the new case RA patients.

The current study has some limitations: First, the sample size examined in this study, especially the sample size of the new case RA patients, was small and may not be indicative of all patients with RA in Kermanshah province. Second, we could not access the synovial fluid specimens of the new case RA patients owing to ethical considerations. Also, investigating the protein expression levels of CCR6 and RORγt, as well as characteristic cytokines secreted from Th17 cells, might be fruitful for better interpreting our results.

## 5 CONCLUSION

In summation, increased expression of CCR6 and RORγt genes may contribute to the attraction and migration of Th17 cells to the sites of inflammation occurring in RA, including the inflamed joints, and also may be associated with RA pathogenesis. Furthermore, considering that all RA patients in our study were newly diagnosed, it can be concluded that CCR6 and RORγt have a key role in early pathological events in RA and may contribute to RA progression and exacerbation. Nonetheless, to confirm our findings, further detailed studies, including assessing the protein expression levels of CCR6 and RORγt, as well as cytokines secreted from Th17 cells, are warranted in the synovial tissues and the inflamed joints of RA patients.

## AUTHOR CONTRIBUTIONS

Seyed Askar Roghani, Ramin Lotfi, and Mahdi Taghadosi contributed to the idea design, checked the final results, and performed the final revision of the manuscript. Seyed Askar Roghani, Ramin Lotfi, Masood Ghasemzade Soroush, Ali Khorasanizadeh, Parisa Feizollahi, and Zahra Mohammadi Kish performed experiments, calculations, and manuscript writing. Shirin Assar, Parviz Soufivand, and Mehran Pournazari as the specialist physicians referred the RA patients for sampling. All authors reviewed and approved the final manuscript for publication.

## CONFLICT OF INTEREST STATEMENT

The authors declare no conflict of interest.

## ETHICS STATEMENT

This study was done in concord with the principles of the Helsinki Declaration. Approval was granted by the Ethics Committee of the Kermanshah University of Medical Sciences (Ethical code: IR.KUMS.REC.1400.175), Kermanshah, Iran. Written informed consent was taken from all individual participants included in the study.

## Data Availability

The data sets generated during and/or analyzed during the present study are available from the corresponding author upon reasonable request.

## References

[1] Müller‐Ladner U , Pap T , Gay RE , Neidhart M , Gay S . Mechanisms of disease: the molecular and cellular basis of joint destruction in rheumatoid arthritis. Nat Clin Pract Rheumatol. 2005;1(2):102‐110. 10.1038/ncprheum0047 16932639

[2] Buch MH , Eyre S , McGonagle D . Persistent inflammatory and non‐inflammatory mechanisms in refractory rheumatoid arthritis. Nat Rev Rheumatol. 2021;17(1):17‐33. 10.1038/s41584-020-00541-7 33293696

[3] Newton J , Harney S , Wordsworth B , Brown M . A review of the MHC genetics of rheumatoid arthritis. Genes Immun. 2004;5(3):151‐157. 10.1038/sj.gene.6364045 14749714

[4] McInnes IB , Schett G . Cytokines in the pathogenesis of rheumatoid arthritis. Nat Rev Immunol. 2007;7(6):429‐442. 10.1038/nri2094 17525752

[5] Szekanecz Z , Kim J , Koch AE . Chemokines and chemokine receptors in rheumatoid arthritis. Semin Immunol. 2003;15(1):15‐21. 10.1016/s1044-5323(02)00124-0 12495637

[6] van Boekel MA , Vossenaar ER , van den Hoogen FH , van Venrooij WJ . Autoantibody systems in rheumatoid arthritis: specificity, sensitivity and diagnostic value. Arthritis Res. 2002;4(2):87‐93. 10.1186/ar395 11879544 PMC128920

[7] Szekanecz Z , Koch AE . Successes and failures of chemokine‐pathway targeting in rheumatoid arthritis. Nat Rev Rheumatol. 2016;12(1):5‐13. 10.1038/nrrheum.2015.157 26607389

[8] Hughes CE , Nibbs RJB . A guide to chemokines and their receptors. FEBS J. 2018;285(16):2944‐2971. 10.1111/febs.14466 29637711 PMC6120486

[9] Elemam NM , Hannawi S , Maghazachi AA . Role of chemokines and chemokine receptors in rheumatoid arthritis. Immunotargets Ther. 2020;9:43‐56. 10.2147/itt.s243636 32211348 PMC7074856

[10] Pournazari M , Feizollahi P , Roghani SA , et al. Increased plasma levels of CCL20 in peripheral blood of rheumatoid arthritis patients and its association with clinical and laboratory parameters. Clin Rheumatol. 2022;41(1):265‐270. 10.1007/s10067-021-05899-x 34477989

[11] Paulissen SMJ , van Hamburg JP , Dankers W , Lubberts E . The role and modulation of CCR6+ Th17 cell populations in rheumatoid arthritis. Cytokine. 2015;74(1):43‐53. 10.1016/j.cyto.2015.02.002 25828206

[12] Korn T , Bettelli E , Oukka M , Kuchroo VK . IL‐17 and Th17 cells. Annu Rev Immunol. 2009;27:485‐517. 10.1146/annurev.immunol.021908.132710 19132915

[13] Tesmer LA , Lundy SK , Sarkar S , Fox DA . Th17 cells in human disease. Immunol Rev. 2008;223:87‐113. 10.1111/j.1600-065X.2008.00628.x 18613831 PMC3299089

[14] Lee AY , Körner H . CCR6 and CCL20: emerging players in the pathogenesis of rheumatoid arthritis. Immunol Cell Biol. 2014;92(4):354‐358. 10.1038/icb.2013.97 24394994

[15] Kochi Y , Okada Y , Suzuki A , et al. A regulatory variant in CCR6 is associated with rheumatoid arthritis susceptibility. Nat Genet. 2010;42(6):515‐519. 10.1038/ng.583 20453841

[16] Tan J , Liu H , Huang M , et al. Small molecules targeting RORγt inhibit autoimmune disease by suppressing Th17 cell differentiation. Cell Death Dis. 2020;11(8):697. 10.1038/s41419-020-02891-2 32829384 PMC7443190

[17] Chi X , Jin W , Zhao X , et al. RORγt expression in mature T(H)17 cells safeguards their lineage specification by inhibiting conversion to T(H)2 cells. Sci Adv. 2022;8(34):eabn7774. 10.1126/sciadv.abn7774 36026450 PMC9417185

[18] Bukhari M , Thomson W , Naseem H , et al. The performance of anti‐cyclic citrullinated peptide antibodies in predicting the severity of radiologic damage in inflammatory polyarthritis: results from the Norfolk Arthritis Register. Arthritis Rheum. 2007;56(9):2929‐2935. 10.1002/art.22868 17763407 PMC2435419

[19] Kay J , Upchurch KS . ACR/EULAR 2010 rheumatoid arthritis classification criteria. Rheumatology. 2012;51(suppl 6):vi5‐vi9. 10.1093/rheumatology/kes279 23221588

[20] Pfaffl MW . A new mathematical model for relative quantification in real‐time RT‐PCR. Nucleic Acids Res. 2001;29(9):e45. 10.1093/nar/29.9.e45 11328886 PMC55695

[21] Inoue E , Yamanaka H , Hara M , Tomatsu T , Kamatani N . Comparison of Disease Activity Score (DAS)28‐erythrocyte sedimentation rate and DAS28‐C‐reactive protein threshold values. Ann Rheum Dis. 2007;66(3):407‐409. 10.1136/ard.2006.054205 16926186 PMC1856019

[22] van Hamburg JP , Asmawidjaja PS , Davelaar N , et al. Th17 cells, but not Th1 cells, from patients with early rheumatoid arthritis are potent inducers of matrix metalloproteinases and proinflammatory cytokines upon synovial fibroblast interaction, including autocrine interleukin‐17A production. Arthritis Rheum. 2011;63(1):73‐83. 10.1002/art.30093 20954258

[23] Jimeno R , Gomariz RP , Garín M , et al. The pathogenic Th profile of human activated memory Th cells in early rheumatoid arthritis can be modulated by VIP. J Mol Med. 2015;93(4):457‐467. 10.1007/s00109-014-1232-4 25430993 PMC4366555

[24] Takatori H , Kanno Y , Chen Z , O'Shea JJ . New complexities in helper T cell fate determination and the implications for autoimmune diseases. Mod Rheumatol. 2008;18(6):533‐541. 10.1007/s10165-008-0099-z 18679768 PMC2596867

[25] Arroyo‐Villa I , Bautista‐Caro MB , Balsa A , et al. Frequency of Th17 CD4+ T cells in early rheumatoid arthritis: a marker of anti‐CCP seropositivity. PLoS One. 2012;7(8):e42189. 10.1371/journal.pone.0042189 22870298 PMC3411698

[26] Murphy PM , Baggiolini M , Charo IF , et al. International union of pharmacology. XXII. nomenclature for chemokine receptors. Pharmacol Rev. 2000;52(1):145‐176. 10699158

[27] Wasilko DJ , Johnson ZL , Ammirati M , et al. Structural basis for chemokine receptor CCR6 activation by the endogenous protein ligand CCL20. Nat Commun. 2020;11(1):3031. 10.1038/s41467-020-16820-6 32541785 PMC7295996

[28] Onuora S . Targeting the CCR6‐CCL20 axis improves experimental PsA. Nat Rev Rheumatol. 2021;17(8):441. 10.1038/s41584-021-00663-6 34226726

[29] Cheng P , Zhang Y , Huang H , et al. Association between CCR6 and rheumatoid arthritis: a meta‐analysis. Int J Clin Exp Med. 2015;8(4):5388‐5396. 26131115 PMC4483986

[30] Hirota K , Yoshitomi H , Hashimoto M , et al. Preferential recruitment of CCR6‐expressing Th17 cells to inflamed joints via CCL20 in rheumatoid arthritis and its animal model. J Exp Med. 2007;204(12):2803‐2812. 10.1084/jem.20071397 18025126 PMC2118525

[31] Razawy W , Asmawidjaja PS , Mus AM , et al. CD4(+) CCR6(+) T cells, but not γδ T cells, are important for the IL‐23R‐dependent progression of antigen‐induced inflammatory arthritis in mice. Eur J Immunol. 2020;50(2):245‐255. 10.1002/eji.201948112 31778214 PMC7028107

[32] Takeuchi T , Miyasaka N , Inui T , et al. High titers of both rheumatoid factor and anti‐CCP antibodies at baseline in patients with rheumatoid arthritis are associated with increased circulating baseline TNF level, low drug levels, and reduced clinical responses: a post hoc analysis of the RISING study. Arthritis Res Ther. 2017;19(1):194. 10.1186/s13075-017-1401-2 28865493 PMC5581496

[33] Kaneko S , Kondo Y , Yokosawa M , et al. The RORγt‐CCR6‐CCL20 axis augments Th17 cells invasion into the synovia of rheumatoid arthritis patients. Mod Rheumatol. 2018;28(5):814‐825. 10.1080/14397595.2017.1416923 29251019

[34] Meitei HT , Jadhav N , Lal G . CCR6‐CCL20 axis as a therapeutic target for autoimmune diseases. Autoimmun Rev. 2021;20(7):102846. 10.1016/j.autrev.2021.102846 33971346

[35] Mangelsdorf DJ , Evans RM . The RXR heterodimers and orphan receptors. Cell. 1995;83(6):841‐850. 10.1016/0092-8674(95)90200-7 8521508

[36] Huh JR , Littman DR . Small molecule inhibitors of RORγt: targeting Th17 cells and other applications. Eur J Immunol. 2012;42(9):2232‐2237. 10.1002/eji.201242740 22949321 PMC3609417

[37] Kondo Y , Yao Z , Tahara M , et al. Involvement of RORγt‐overexpressing T cells in the development of autoimmune arthritis in mice. Arthritis Res Ther. 2015;17(1):105. 10.1186/s13075-015-0606-5 25928901 PMC4436146

